# Campylobacter infections expected to increase due to climate change in Northern Europe

**DOI:** 10.1038/s41598-020-70593-y

**Published:** 2020-08-17

**Authors:** Katrin Gaardbo Kuhn, Karin Maria Nygård, Bernardo Guzman-Herrador, Linda Selje Sunde, Ruska Rimhanen-Finne, Linda Trönnberg, Martin Rudbeck Jepsen, Reija Ruuhela, Wai Kwok Wong, Steen Ethelberg

**Affiliations:** 1 Infectious Disease Epidemiology and Prevention, Statens Serum Institut, Artillerivej 5, Copenhagen, Denmark; 2 Department of Infectious Disease Epidemiology, Norwegian Institute of Public Health, Oslo, Norway; 3 Department of Health Security, National Institute for Health and Welfare, Helsinki, Finland; 4 Department of Monitoring and Evaluation, Public Health Agency of Sweden, Solna, Sweden; 5 Section for Geography, IGN, University of Copenhagen, Copenhagen, Denmark; 6 Weather and Climate Change Impact Research, Finnish Meteorological Institute, Helsinki, Finland; 7 Department of Hydrology, Norwegian Water Resources and Energy Directorate, Oslo, Norway; 8 Global Health Section, Department of Public Health, University of Copenhagen, Copenhagen, Denmark

**Keywords:** Epidemiology, Climate-change impacts, Projection and prediction

## Abstract

Global climate change is predicted to alter precipitation and temperature patterns across the world, affecting a range of infectious diseases and particularly foodborne infections such as *Campylobacter*. In this study, we used national surveillance data to analyse the relationship between climate and campylobacteriosis in Denmark, Finland, Norway and Sweden and estimate the impact of climate changes on future disease patterns. We show that *Campylobacter* incidences are linked to increases in temperature and especially precipitation in the week before illness, suggesting a non-food transmission route. These four countries may experience a doubling of *Campylobacter* cases by the end of the 2080s, corresponding to around 6,000 excess cases per year caused only by climate changes. Considering the strong worldwide burden of campylobacteriosis, it is important to assess local and regional impacts of climate change in order to initiate timely public health management and adaptation strategies.

## Introduction

The zoonotic pathogen *Campylobacter* is the most commonly reported cause of human bacterial gastroenteritis throughout Europe, including the Nordic countries (Denmark, Finland, Iceland, Norway and Sweden). Since 2008, the reported incidence of *Campylobacter* infections in Europe has increased and is now three times greater than that of salmonellosis^1^. Domestic cases of campylobacteriosis are commonly linked to consumption of contaminated food or drink such as poultry, unpasteurized milk and cross-contaminated vegetables^2–4^. Lately environmental and behavioural factors such as recreational water contact, occupational exposure (e.g. poultry farms and abattoirs) and contact to household pets^4–6^ have also emerged as important *Campylobacter* transmission routes. Infection rates are highest in children under five and young adults, and in all temperate regions, particularly the Nordic countries, the incidence of disease varies seasonally and tends to peak during the late spring and summer months^7,8^. This peak may represent a combination of fluctuating infection rates in poultry^9^ (caused either by a direct impact of temperature on *Campylobacter* growth rates or indirect effects such as changes in contamination sources) and increased human exposure to environmental reservoirs as well as different eating and hygiene patterns during the summer months^4,8,10^.

Global climate change is predicted to alter temperatures and precipitation across the world with strong direct and indirect impacts on human health^11,12^. Because weather and climate are important determinants of infectious diseases, including food-borne, it is relevant and important to estimate future changes in disease patterns related to climate or environmental changes^11^. Many published reports highlight a strong effect of climate changes on important infectious diseases such as malaria, West Nile virus, cholera, and tuberculosis^13–17^, but—considering their overall contribution to the global disease burden—the impact of climate changes on zoonotic diseases has been less well studied. For instance, it is predicted that climate changes are likely to increase the future incidence of salmonellosis in selected areas^18–20^, but evidence of a link between climate and *Campylobacter* is conflicting and of varying strength^21–25^. Generally, it appears that human campylobacteriosis—not considering the seasonal variation—is driven by a complex mixture of climatic drivers where precipitation in particular may be important^21,24,26,27^. This is especially highlighted for extreme rainfall^28^ which has been linked to large *Campylobacter* outbreaks^7,29–34^. Although campylobacteriosis is rarely fatal, the global disease burden—particularly from an economic perspective—is currently high^35–37^ and, irrespective of climate, predicted to increase in the future^38,39^. This natural increase, reflecting changing populations, exposure patterns and microbiological features such as antimicrobial resistance, combined with climatic changes, can result in a future heavier disease burden and higher costs to societies. Establishing how extreme weather events and climate changes affect campylobacteriosis can form the basis of well-guided early warning systems in vulnerable areas and better targeting of prevention and control measures, potentially reducing the public health and economic impact of *Campylobacter* in these areas.

In this study, we analyse the temporal and spatial relationship between climatic factors and the incidence of *Campylobacter* in the Nordic countries by fitting observed reported disease cases and weather events in a Poisson regression model and applying these relationships to estimate the future incidence of disease associated with projected climate changes.

## Results

### Campylobacter and climate at baseline 2000–2015

A total of 64,034 reported cases of *Campylobacter* were included in the final database. The average number of cases per municipality during the study period was 154 (median: 0 and maximum: 274). During the baseline period from 2000 to 2015, the average annual number of *Campylobacter* cases per 100,000 persons in the four countries was 42, ranging from 25 in Norway to 60 in Denmark.

For all locations, the average weekly temperature observed during the study period was 4.4 °C (ranging from − 35.5 to 32.8 °C) and the average daily precipitation was 2.2 mm (ranging from 0 to 105 mm). Overall, each municipality experienced a total of 2.4 heat waves and 3.3 heavy precipitation events (excluding snow) during the study period. Fitting the six regression models on the 10% of omitted data showed that the number of *Campylobacter* cases was best predicted using the standard Poisson regression model for both winter and summer periods with a time-lag of 1 week (i.e. using the previous week’s climate data to predict the following week’s cases of *Campylobacter*). The models predicted 79.7 and 84.5% data records correctly in the winter and summer months, respectively (data not shown). The final models for *Campylobacter* and climate showed that the number of *Campylobacter* cases in any week during the summer increased significantly with increasing temperature, precipitation and number of heavy precipitation events in the previous week (Table 1). Conversely, an increase in the number of heat waves in any week during the summer as well as increases in precipitation during the winter months decreased the number of *Campylobacter* cases reported 1 week later (Table 1).

**Table 1:** Models for the relationship between weather and variations in *Campylobacter* cases.

Variable	Coefficient	95% CI	p	Overall statistics
**Summer period model (April–September)**				N. obs = 78,842; X^2^ = 24,036; p < 0.001; R^2^ = 0.51
Temperature	0.09	0.09–0.10	< 0.001	
Precipitation	0.30	0.29–0.32	< 0.001	
Heat wave	− 0.10	− 0.15 to − 0.05	< 0.001	
Heavy precipitation	0.79	0.76–0.82	< 0.001	
**Winter period model (October–May)**				N. obs = 66,648; x^2^ = 18,235; p < 0.001; R^2^ = 0.33
Temperature	0.18	0.17–0.18	< 0.001	
Precipitation	− 0.18	− 0.19 to − 0.18	< 0.001	
Heavy precipitation	− 0.05	− 0.07 to − 0.03	< 0.001	

Explanatory variables are related to the outcome with a 1 week time-lag (the weather in 1 week determines the following week’s number of cases).

To predict the number of *Campylobacter* cases in 2000–2015, the Poisson models were applied to observed climate data from 2000–2015 (separately for winter and summer months). We used the resulting predictions as the disease baseline. Compared to observed weekly number of cases at municipality level during 2000–2015, these predictions were overall 93.3% accurate (data not shown). We estimated the effects of arbitrary climate changes in our models by varying the different explanatory variables (Table 2). For instance, a 1 mm increase in precipitation (all other variables remaining unchanged) in any municipality in any week during the summer will result in a 38% increase in the number of *Campylobacter* cases in that municipality the following week.

**Table 2:** Quantitative translation of weather impact on *Campylobacter* cases in any municipality in the Nordic countries.

Scenario	Number of cases	Additional cases	% change
Normal	16	n.a.	n.a.
1 °C increase in mean temperature	18	2	13
1 mm increase in precipitation during the summer	22	6	38
One additional heat wave^a^ in the summer	14	-2	-13
One additional heavy precipitation^b^ event in the summer	24	8	50

The hypothetical situation where one scenario event takes place in any municipality in a given week, and the resulting number of (additional) Campylobacter cases the following week because of this event.

^a^Heat wave: three consecutive days with temperatures exceeding 99th percentile of the daily maximum temperature from 2000 to 2015.

^b^Heavy precipitation: a day with precipitation exceeding 95th percentile of daily precipitation from 2000 to 2015.

### Predicting *Campylobacter* in the future using climate change projections

**Figure 1:**
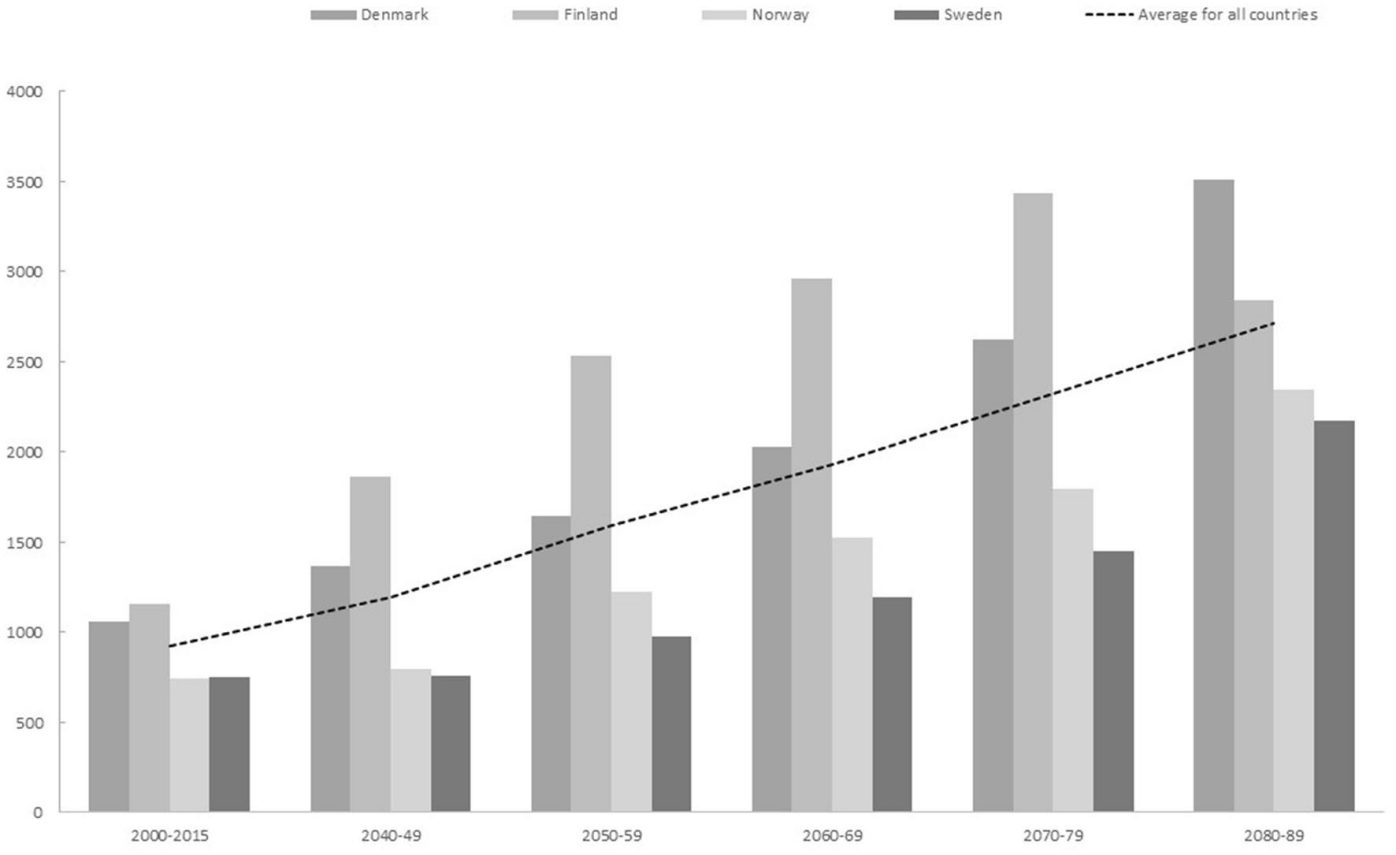
Number of *Campylobacter* cases estimated at baseline and predicted for future scenarios.

Our predictions indicate that *Campylobacter* cases in the four Nordic countries combined can increase by 25% by the end of the 2040s and 196% by the end of the 2080s compared to the predicted baseline of 2000–2015 (Fig. 1). These impacts vary with country and time period with the highest increases predicted in Denmark and Norway during the late part of the time period (Fig. 1).

**Figure 2:**
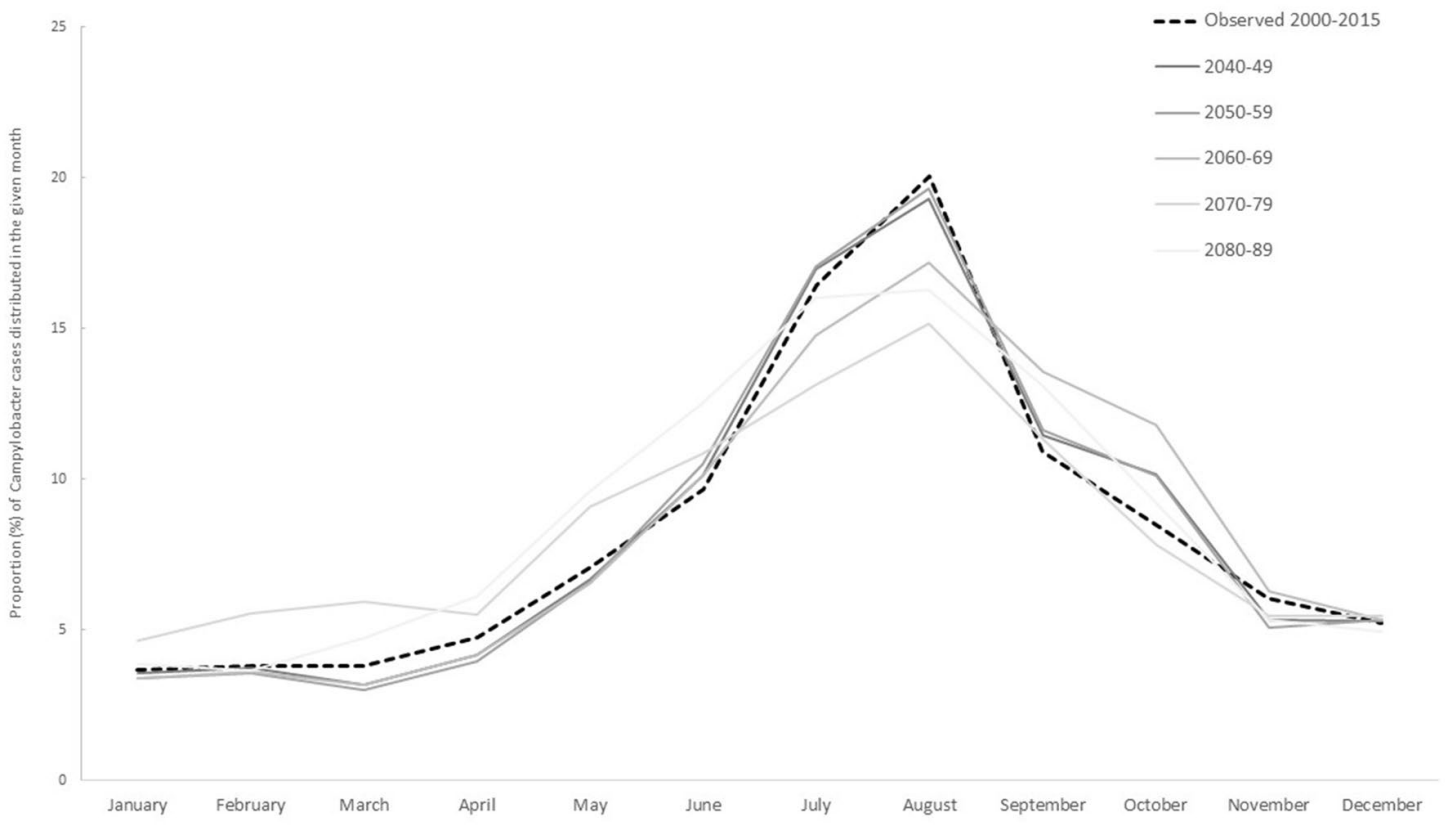
Seasonal distribution of *Campylobacter* cases observed at baseline and predicted for the future.

Our models also predict a change in the future seasonal distribution of cases. At present, *Campylobacter* cases increase during the spring and summer and almost half of the annual total cases are reported between July and September alone (Fig. 2). During 2040–2059, this pattern will remain similar although the high seasons extends until November. For the later scenarios, the seasonal variation has become less pronounced with the number of cases increasing from April and remaining higher until November. Here, only 32% of cases will be reported in July–September.

**Figure 3:**
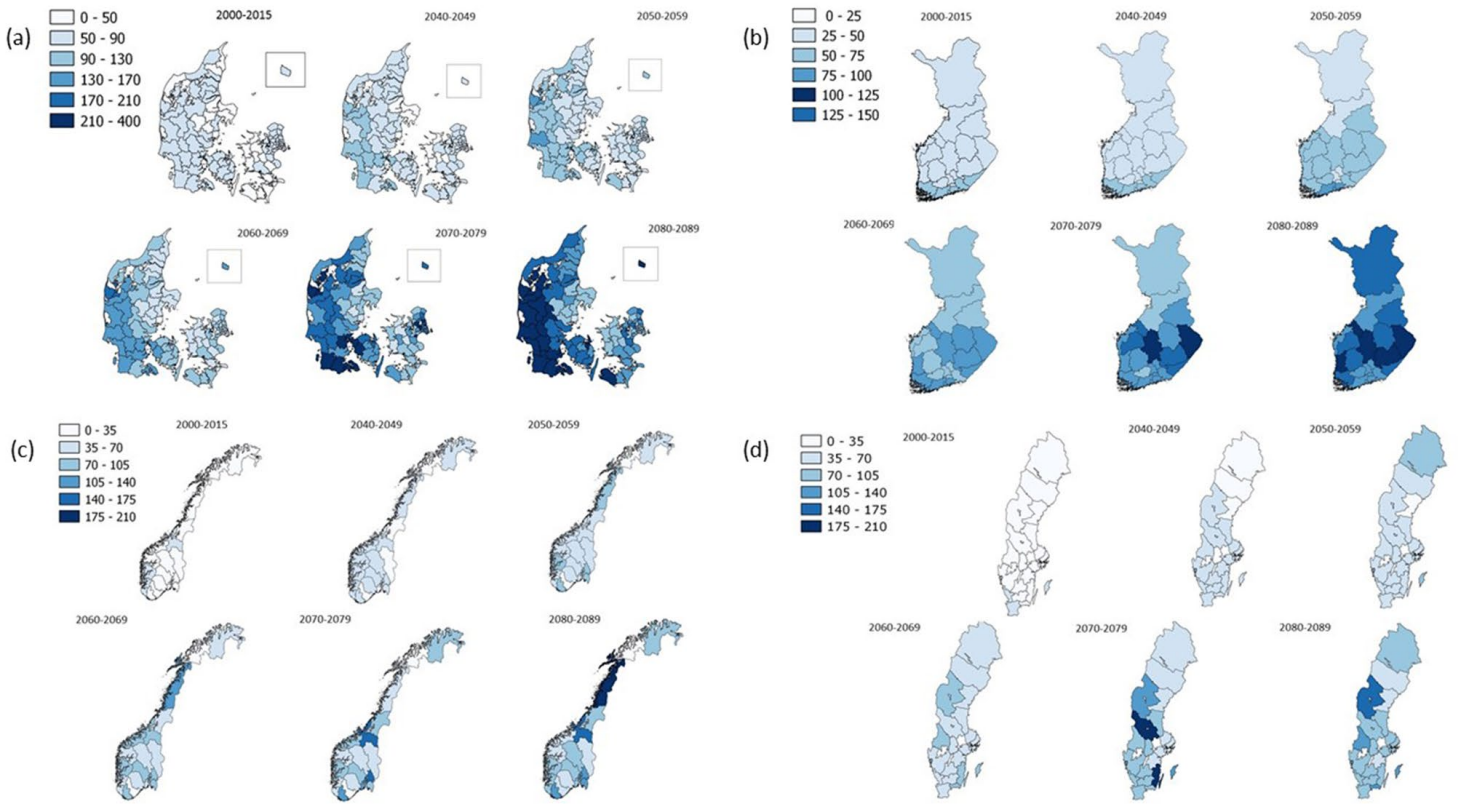
Predicted *Campylobacter* incidences in (**a**) Denmark, (**b**) Finland, (**c**) Norway and (**d**) Sweden, 2000–2090.

Using estimations of future population sizes, we translated the predicted number of cases to incidences (Table 3), showing that the average annual incidence of *Campylobacter* per 100,000 persons is expected to increase from 42 in 2000–2015 to 117 in 2080–2089 (Table 3). These results were displayed at regional or municipality level, showing that, in Denmark, incidences will increase more in western municipalities (Fig. 3a). In Finland, the central regions are predicted to experience the highest increases in *Campylobacter* (Fig. 3b), while in Norway, the strongest changes are in central and northern regions (Fig. 3c). For Sweden, the overall changes are less pronounced but some central and southern regions are still predicted to experience increases in the incidence of *Campylobacter* (Fig. 3d).

**Table 3:** Average annual *Campylobacter* incidence and total number of excess cases observed at baseline and predicted for future decades.

Country	2000–2015	2040–2049		2050–2059		2060–2069		2070–2079		2080–2089	
	Incidence	Incidence	Excess cases	Incidence	Excess cases	Incidence	Excess cases	Incidence	Excess cases	Incidence	Excess cases
Denmark	60	72	94	85	294	103	590	164	1,092	170	1884
Finland	45	45	195	58	795	71	1,150	94	1547	107	876
Norway	25	45	446	58	956	70	1,318	82	1635	97	2,216
Sweden	31	41	− 157	54	-9	64	141	89	315	93	960
Average	42	51	145	64	509	77	800	107	1,147	117	1,484

Number of cases/100,000 population.

Compared to the expected number of cases during the time period.

By subtracting the expected number of cases due to ‘natural variation’ in 2040–2089 from the climate-modelled number of cases, we calculated the excess number of cases caused by climate changes alone (i.e. how many cases in the future are caused by climate only and not by natural variation in the form of demographic changes, microbiology, diagnostic practices etc.). These results show that climate changes alone can result in an average 145 excess annual cases of *Campylobacter* by 2040–2049 and almost 1,500 by the end of the 2080s in each country per year (Table 3). The effect varies with country, with less pronounced effects particularly in Sweden (Table 3).

## Discussion

Using national surveillance data collected from four Nordic countries, we investigated the relationship between the temporal and geographical distribution of *Campylobacter* and climate factors and, based on these relationships, estimated a future scenario for the likely effect of predicted climate changes on campylobacteriosis.

Our models show that the temporal and geographical distribution of *Campylobacter* in Denmark, Finland, Norway and Sweden can be associated with temperature and precipitation. Specifically, increases in temperature and heavy rainfall may increase the number of *Campylobacter* cases reported the following week. On the other hand, according to our models, heat waves and winter precipitation (i.e. both rain and snowfall) may decrease the number of cases reported. Generally, these results support the theory that transmission via the environment could be important for cases which are not explained by consumption or handling of poultry^3,4,6^. People in the Nordic countries are weather dependent and spend an increasing amount of time outdoors when weather conditions are suitable, resulting in a higher likelihood of contact to environmental infection sources. At the same time, heavy rainfall can mobilise and distribute bacteria, facilitating transmission from contact to water and mud rather than dry surfaces^40,41^.

Our predicted climate changes were based on projections from the EUR-11 HIRHAM-5 RCM for the RCP8.5 scenario^42,43^. According to these, by the year 2,100, average annual temperatures in the Nordic countries may increase by an average of 4.2 °C, ranging from 3.5 °C in the southernmost parts to 5 °C in northern and central areas. Precipitation is predicted to increase by 25% with only small variations between the countries, and heavy precipitation events can increase by up to 45% across the region. The frequency of heat waves in this region is also affected by climate changes, with an overall 20% increase predicted for most areas (but up to 45% in some parts of Norway).

By incorporating these climate changes into our models, we estimate an overall increase in campylobacteriosis of almost 200% by the end of this century. This translates to nearly 6,000 excess *Campylobacter* cases per year in these four countries which could potentially be linked only to climate changes. Campylobacteriosis is a highly seasonal disease in the Nordic countries, and our models also indicate a future change in the seasonality of transmission. Rather than the current large increase limited to the summer months, the future pattern can show a less pronounced peak during the summer but greater increases earlier in the year, i.e. a prolonged *Campylobacter* season with higher incidences over longer time.

Only few published studies have analysed the association between climate and *Campylobacter* cases—and some with conflicting results. Generally, it appears that *Campylobacter* in chicken flocks fluctuates with temperature and humidity^9,44^ while incidences in humans may be linked to fluctuations in both temperature and rainfall and especially heavy precipitation events^21,24,25,28,45–48^. Heat waves have been linked to reduced incidences of *Campylobacter*^21,22^. Projections of future patterns in campylobacteriosis related to climate changes are also few in the published literature, but available results suggest increases of between 3 and 20% by the end of the century in different geographical locations^21,46,49,50^. The results from this study of *Campylobacter* in Scandinavia to some extent confirm these findings while also providing further evidence of a climate dependence. The increases predicted as a result of climate changes are higher than from other studies, however this is expected as the methods, data resolution, geographical areas, demographic and climate change projections are all different.

Given the data available, the results presented here should be considered with respect to several limitations. Our models predicted present levels of campylobacteriosis with an overall accuracy of more than 90%. That they are not more accurate most likely reflects underlying variations in data quality, both with respect to disease and climate data, as well as the fact that other unaccounted factors play different roles in different locations. An important limitation is that disease data have been collected using different methods and different definitions in the four countries. Firstly, our database of observed *Campylobacter* cases from 2000 to 2015 included only domestic cases for Norway and Sweden but both domestic and cases of unknown origin from Denmark and Finland. Assuming that some of these unknown cases were in fact acquired abroad, this may have clouded the association between climate and disease patterns. The ‘time’ variable is also particularly important. The date used for disease data refers to the week in which the sample was taken (Norway and Finland) or in which the sample was received in the diagnostic laboratory (Denmark and Sweden)—and the time-lag between actual symptom onset and these dates is unknown (presumably several days). Therefore, it is difficult to establish a true infection date estimate. Further, the climate data were aggregated from daily to weekly data, which reduces data size but also the likelihood of capturing variations. Spatially, there was a mismatch between the disease data collected at relatively high resolution (down to point-level) and then aggregated to municipality level, and climate data at low resolution (25 × 25 km), which reduced the geographical correlation between disease and climate data and the ability of the models to capture high-resolution variations. This is especially important for extreme weather events, which often occur as well-confined local events. Also, it is likely that for some cases, the exposure leading to infection did not occur at the reported geographical location of each cases. Again, this limitation reduces the chance of catching the association between local weather events which could have affected the risk of exposure. Worth noting is also that we simulated climate changes using outputs from one model and one emission scenario only, limiting the predictions to a single situation rather than a range of different ones. However, this is unlikely to have impacted the general accuracy of the predictions as the both the EUR-11 HIRHAM-5 model and the RCP8.5 scenario are considered robust and often used for similar scientific purposes. Overall, our predictions were made using a relatively simple Poisson model (see Supplementary Methods), and the results should also be considered with reference to these specific modelling constraints. The underlying assumptions for linear and Poisson regressions, particularly the assumption of linearity and equal mean and variance, may not be universally valid in the dataset used (i.e. for different geographical locations). Standard Poisson regression may in this case not be optimal for investigating climate/disease relationships, and more sophisticated dynamic models should also be assessed.

In the context of exploring linkages between climate and disease, it is important to note that many such associations are likely to be indirect. For *Campylobacter* in particular, disease transmission reflects chicken flock infection rates and human behaviour (barbecues, outdoor activities) both of which are also strongly dependent on weather and therefore likely to be altered in a changing climate. In addition, disease incidences are also determined by the structure and function of the socio-economic and public health systems which, given different constraints, may also appear different in the future. In relation to this, our results likely over-estimate the future number of cases as public health systems will adapt to higher incidences by taking stronger measures to reduce the incidence. Finally, because *Campylobacter* is a zoonotic infection, in order to understand disease patterns in the present and future, it is necessary to adopt a One Health approach where evidence and knowledge from the public health, food safety, veterinary and environmental sectors are considered together.

To our knowledge, this is one of the first attempts to describe an association between campylobacteriosis and climate factors using high-quality routinely collected surveillance data and modelling the effect of climate changes on this disease at local and national levels. Overall, the results from our models correlate with published evidence for a *Campylobacter*-climate association. Considering their limitations, the models show that climate changes—particularly increases in rainfall and rainfall intensity—could potentially lead to an increase in the incidence of *Campylobacter* in the Nordic countries. Considering the strong burden of campylobacteriosis across the world, the effects of climate changes on this disease are important to further assess for policy makers to identify potentially vulnerable areas as well as future strategies for public health management and adaptation measures.

## Methods

Denmark, Finland, Norway and Sweden constitute part of the Nordics, a 7-country region in Northern Europe. In 2015, approximately 26 million people inhabited these four countries in a total of 1,121 municipalities. Human *Campylobacter* infection is under statutory surveillance in all four countries where clinicians or laboratories must notify a confirmed case to national public health authorities: Statens Serum Institut in Denmark (through the national microbiology database, MiBa), the National Institute for Health and Welfare in Finland, The Norwegian Institute of Public Health (through the Norwegian Surveillance System for Communicable Diseases, MSIS) and the Public Health Agency of Sweden (in the national surveillance system SmiNet). The laboratory criteria for a *Campylobacter* diagnosis is similar across the countries: isolation of *Campylobacter* spp. by culture from faeces or blood. Since 2013, the Swedish criteria have also included samples positive for *Campylobacter* using direct PCR on stool samples. During the study period, PCR diagnosis was not routinely established in the other countries. Each case notification contains as a minimum the patient’s name, age and sex, geographical location (address) and a date corresponding to sample taken/sample analysed/diagnosis/registration. For this study, we used *Campylobacter* infections notified in Denmark, Finland and Sweden from 1st January 2000 until 31st December 2015 and Norway from 1st January 2000 until 31st December 2014^51^. For Sweden and Norway, we only included “domestically acquired” infections (i.e. excluded travel acquired infections or infections with unknown origin). For Denmark and Finland, we included both domestic infections and those of unknown origin as the proportion of unknown infections was more than 80%^51^ and the exclusion of these would have resulted in a significant loss of data. The disease data were aggregated to total number of cases per week per municipality for the study period.

Baseline climate data from 2000 to 2015 at 0.25° × 0.25° resolution (approximately 25 × 25 km) for all four countries were extracted from the E-OBS daily gridded dataset developed by the European Climate Assessment & Dataset project^52^. The climate data were aggregated to municipality level using zonal statistics in a Geographical Information System (GIS). From the daily data, for each municipality, we calculated weekly average temperature, precipitation, number of heat waves and number of heavy precipitation events. Heavy precipitation was defined as a day with precipitation exceeding the 95th percentile of daily precipitation using as baseline the municipal average from 2000–2015 (only days with precipitation > 1 mm were considered). In order to explore how precipitation patterns and associations varied throughout the year, we calculated precipitation for both ‘winter’ (October–March) and ‘summer’ months (April–September). A heat wave was defined as three consecutive days in which the temperature exceeded the 99th percentile of the daily maximum temperature using as baseline the municipal average from 2000 to 2015. The definition of heat waves was applied only for the summer months (April–September).

The final database of disease and climate included per municipality per week and year from 2000 to 2015: number of reported *Campylobacter* cases, precipitation and temperature, the number of heat waves and the number of days with heavy precipitation. A total of 163,997 data records were included in the database, representing 416 municipalities. Of these, a random sample of 10% (16,400 records) were omitted from the initial data analysis and used to validate the predictive fit of the final model.

Future climate change scenarios for the period 2040–2089 for all four countries were projected using bias-corrected^53^ outputs from the EUR-11 HIRHAM-5 regional atmospheric climate model (RCM) with a resolution of 12.5 km, generated by the EURO-CORDEX ensemble and based on the latest scenarios defined by the Intergovernmental Panel on Climate Change (IPCC)^42^. We used the RCP8.5 emission ‘worst case business as usual’ scenario corresponding to a high increasing greenhouse gas emissions pathway, not including specific climate mitigation targets^54^. We chose this scenario because observed climatic changes correspond to those predicted by the RCP8.5 scenario^12,55^. Absolute daily values for temperature and precipitation in 2040–8989 for all municipalities were calculated by comparing the EUR-11 projections to the municipal average from 2000 to 2015. From these, the weekly average temperature, precipitation, number of heat waves and number of heavy precipitation events at municipality level were calculated and finally aggregated to ten-year averages at a weekly basis.

Data were explored using descriptive statistics. We modelled the relationship between *Campylobacter* cases and climate using three approaches: standard Poisson regression, zero-inflated Poisson regression and standard Poisson regression omitting all observations with zero *Campylobacter* cases. These approaches were chosen to identify which model best fitted the observed data, considering the large amount of zero counts in the data. Time-lags were investigated to identify the optimal interval between when a *Campylobacter* case was reported and when the climate variables were measured. The models were constructed using a backwards stepwise multivariate approach where the full model containing all explanatory variables was run and the least significant variables removed one by one until only significant variables remained. The final model was adjusted for the effect of week (season), year (changing notification rates) and municipality (geographical variation) and for interaction between the climate variables. Separate models were constructed for the winter (October–March) and summer (April–September) periods to account for the effect of precipitation and temperatures varying throughout the year. Model predictive fits were evaluated using the 10% of data records omitted from the analysis. The models and underlying assumptions are described in detail in the Supplementary Methods.

The best fit final models were applied to the baseline climate data and the EUR-11 climate change scenario data, resulting in the estimated number of average cases per municipality per week for 2000–2015 and in ten-year intervals from 2040 to 2089. We compared the weekly predicted number of cases per municipality for the baseline of 2000–2015 to the observed number in order to estimate the predictive accuracy (in percent) of the models. We converted the future predicted number of *Campylobacter* cases per municipality or region to future incidences per region/municipality using projected population sizes obtained from the National Statistical Offices in each country.

The excess number of *Campylobacter* cases, caused by climate change alone (i.e. adjusted for ‘natural variation’), was calculated firstly by estimating the expected monthly and annual average number of cases per municipality for 2040–2089 (natural variation) using the average proportional change in the number of cases for each municipality from year to year during the baseline 2000–2015, assuming similar variability for the years 2040–2089. In order to calculate excess cases, we then subtracted these from the number of cases predicted from the initial climate change scenario modelling.

Data were analysed using the STATA software 14.0 (Statacorp., Lakeway, TX, USA) and GIS analyses undertaken using QGIS 2.14.11. (QuantumGIS, Open Source Geospatial Federation Project, https://www.qgis.org).

### Ethical considerations

This study was approved under the general agreement for non-interventional database studies between the Danish Data Protection Agency and Statens Serum Institut, reference number 2008-54-0474. According to Danish regulations, ethical committee approval is not required for studies which do not involve analysis of biological material from human subjects. The use of national surveillance data does not require informed consent from subjects.

## Supplementary information

Supplementary file1

## Data Availability

The data generated and analysed during the current study are not publicly available because they are classified as sensitive surveillance data, but are available from the corresponding author on reasonable request. All methods were carried out in accordance with relevant guidelines and regulations.

## References

[1] European Food Safety Authority & European Centre for Disease Prevention and Control (2016). The European Union summary report on trends and sources of zoonoses, zoonotic agents and food-borne outbreaks in 2015. EFSA J..

[2] Boysen L (2014). Source attribution of human campylobacteriosis in Denmark. Epidemiol. Infect..

[3] MacDonald E (2015). Risk factors for sporadic domestically acquired campylobacter infections in Norway 2010–2011: A national prospective case-control study. PLoS ONE.

[4] Kuhn KG, Nielsen EM, Mølbak K, Ethelberg S (2018). Determinants of sporadic Campylobacter infections in Denmark: A nationwide case–control study among children and young adults. Clin. Epidemiol..

[5] Gras LM (2013). Increased risk for *Campylobacter jejuni* and *C. coli* infection of pet origin in dog owners and evidence for genetic association between strains causing infection in humans and their pets. Epidemiol. Infect..

[6] Ravel A, Pintar K, Nesbitt A, Pollari F (2016). Non food-related risk factors of campylobacteriosis in Canada: A matched case–control study. BMC Public Health.

[7] Kuhn KG, Nielsen EM, Mølbak K, Ethelberg S (2018). Epidemiology of campylobacteriosis in Denmark 2000–2015. Zoonoses Public Health.

[8] Strachan NJC (2013). Identifying the seasonal origins of human campylobacteriosis. Epidemiol. Infect..

[9] Patrick ME (2004). Effects of climate on incidence of *Campylobacter* spp. in humans and prevalence in broiler flocks in Denmark. Appl. Environ. Microbiol..

[10] David JM (2017). Do contamination of and exposure to chicken meat and water drive the temporal dynamics of Campylobacter cases?. Epidemiol. Infect..

[11] McMichael AJ, Woodruff RE, Hales S (2006). Climate change and human health: present and future risks. Lancet Lond. Engl..

[12] Watts N (2018). The Lancet Countdown on health and climate change: From 25 years of inaction to a global transformation for public health. Lancet.

[13] Baylis M (2017). Potential impact of climate change on emerging vector-borne and other infections in the UK. Environ. Health Glob. Access Sci. Source.

[14] Murdock CC, Sternberg ED, Thomas MB (2016). Malaria transmission potential could be reduced with current and future climate change. Sci. Rep..

[15] Semenza JC (2016). Climate change projections of West Nile virus infections in Europe: Implications for blood safety practices. Environ. Health Glob. Access. Sci. Source.

[16] Walker JT (2018). The influence of climate change on waterborne disease and Legionella: A review. Perspect. Public Health.

[17] Zhang CY, Zhang A (2019). Climate and air pollution alter incidence of tuberculosis in Beijing, China. Ann. Epidemiol..

[18] Akil L, Ahmad HA, Reddy RS (2014). Effects of climate change on Salmonella infections. Foodborne Pathog. Dis..

[19] Jiang C (2015). Climate change, extreme events and increased risk of salmonellosis in Maryland, USA: Evidence for coastal vulnerability. Environ. Int..

[20] Semenza JC (2012). Climate change impact assessment of food- and waterborne diseases. Crit. Rev. Environ. Sci. Technol..

[21] Kovats RS (2005). Climate variability and campylobacter infection: An international study. Int. J. Biometeorol..

[22] Milazzo A (2017). The effects of ambient temperature and heatwaves on daily Campylobacter cases in Adelaide, Australia, 1990–2012. Epidemiol. Infect..

[23] Tam CC, Rodrigues LC, O’Brien SJ, Hajat S (2006). Temperature dependence of reported Campylobacter infection in England, 1989–1999. Epidemiol. Infect..

[24] Weisent J, Seaver W, Odoi A, Rohrbach B (2014). The importance of climatic factors and outliers in predicting regional monthly campylobacteriosis risk in Georgia, USA. Int. J. Biometeorol..

[25] Yun J (2016). Association between the ambient temperature and the occurrence of human Salmonella and Campylobacter infections. Sci. Rep..

[26] Louis VR (2005). Temperature-driven campylobacter seasonality in England and Wales. Appl. Environ. Microbiol..

[27] Semenza JC (2016). Determinants and drivers of infectious disease threat events in Europe. Emerg. Infect. Dis..

[28] Soneja S (2016). Extreme precipitation events and increased risk of campylobacteriosis in Maryland, USA. Environ. Res..

[29] Auld H, MacIver D, Klaassen J (2004). Heavy rainfall and waterborne disease outbreaks: the Walkerton example. J. Toxicol. Environ. Health A.

[30] Thomas KM (2006). A role of high impact weather events in waterborne disease outbreaks in Canada, 1975–2001. Int. J. Environ. Health Res..

[31] Nichols G, Lane C, Asgari N, Verlander NQ, Charlett A (2009). Rainfall and outbreaks of drinking water related disease and in England and Wales. J. Water Health.

[32] Guzman-Herrador, B. *et al.* Waterborne outbreaks in the Nordic countries, 1998–2012. *Euro Surveill. Bull. Eur. Sur Mal. Transm. Eur. Commun. Dis. Bull.* **20** (2015). 10.2807/1560-7917.es2015.20.24.21160 26111239

[33] Harder-Lauridsen NM, Kuhn KG, Erichsen AC, Mølbak K, Ethelberg S (2013). Gastrointestinal illness among triathletes swimming in non-polluted versus polluted seawater affected by heavy rainfall, Denmark, 2010–2011. PLoS ONE.

[34] Kuhn KG (2017). Epidemiological and serological investigation of a waterborne *Campylobacter jejuni* outbreak in a Danish town. Epidemiol. Infect..

[35] Gibney KB, O’Toole J, Sinclair M, Leder K (2014). Disease burden of selected gastrointestinal pathogens in Australia, 2010. Int. J. Infect. Dis..

[36] Havelaar AH (2012). Disease burden of foodborne pathogens in the Netherlands, 2009. Int. J. Food Microbiol..

[37] Havelaar AH (2015). World Health Organization Global Estimates and Regional Comparisons of the Burden of Foodborne Disease in 2010. PLOS Med..

[38] de Noordhout CM (2015). Current and future Disability-Adjusted Life Years (DALYs) of Salmonella and Campylobacter in Belgium. Arch. Public Health.

[39] de Noordhout CM (2017). Burden of salmonellosis, campylobacteriosis and listeriosis: A time series analysis, Belgium, 2012 to 2020. Eurosurveillance.

[40] Zeigler M (2014). Outbreak of campylobacteriosis associated with a long-distance obstacle adventure race-Nevada, October 2012. MMWR Morb. Mortal. Wkly. Rep..

[41] Stuart TL (2010). Campylobacteriosis outbreak associated with ingestion of mud during a mountain bike race. Epidemiol. Infect..

[42] Jacob D (2014). EURO-CORDEX: New high-resolution climate change projections for European impact research. Reg. Environ. Change.

[43] Kotlarski S (2014). Regional climate modeling on European scales: A joint standard evaluation of the EURO-CORDEX RCM ensemble. Geosci. Model Dev..

[44] Sandberg M (2015). Risk factors for Campylobacter colonization in Danish broiler flocks, 2010 to 2011. Poult. Sci..

[45] Sanderson RA (2018). Spatio-temporal models to determine association between Campylobacter cases and environment. Int. J. Epidemiol..

[46] Sterk A, Schijven J, de Nijs T, Husman AMR (2013). Direct and indirect effects of climate change on the risk of infection by water-transmitted pathogens. Environ. Sci. Technol..

[47] Djennad A (2019). Seasonality and the effects of weather on Campylobacter infections. BMC Infect. Dis..

[48] Lake IR (2019). Exploring Campylobacter seasonality across Europe using The European Surveillance System (TESSy), 2008 to 2016. Eurosurveillance.

[49] Cullen E (2009). The impact of climate change on the future incidence of specified foodborne diseases in Ireland. Epidemiology.

[50] McBride G, Tait A, Slaney D (2014). Projected changes in reported campylobacteriosis and cryptosporidiosis rates as a function of climate change: A New Zealand study. Stoch. Environ. Res. Risk Assess..

[51] Kuhn KG (2019). Campylobacteriosis in the Nordic countries from 2000 to 2015: Trends in time and space. Scand. J. Public Health..

[52] van den Besselaar EJM, Haylock MR, van der Schrier G, Klein Tank AMG (2011). A European daily high-resolution observational gridded data set of sea level pressure. J. Geophys. Res..

[53] Dosio A, Paruolo P (2011). Bias correction of the ENSEMBLES high-resolution climate change projections for use by impact models: Evaluation on the present climate. J. Geophys. Res. Atmos..

[54] Riahi K (2011). RCP 8.5—A scenario of comparatively high greenhouse gas emissions. Clim. Change.

[55] Brown PT, Caldeira K (2017). Greater future global warming inferred from Earth’s recent energy budget. Nature.

