# Supplementary material for: Campylobacter infections expected to increase due to climate change in Northern Europe
Source: Sci Rep. 2020 Aug 17;10:13874. doi: 10.1038/s41598-020-70593-y (PMC7431569; doi:10.1038/s41598-020-70593-y)
Supplement: Supplementary file 1 — Supplementary file1 [file 41598_2020_70593_MOESM1_ESM.docx]

**Campylobacter infections expected to increase due to climate change in Northern Europe**

Katrin Gaardbo Kuhn^1*^, Karin Maria Nygård^2^, Bernardo Guzman-Herrador^2^, Linda Selje Sunde^2^, Ruska Rimhanen-Finne^3^, Linda Trönnberg^4^, Martin Rudbeck Jepsen^5^, Reija Ruuhela^6^, Wai Kwok Wong^7^ and Steen Ethelberg^1,8^

1. *Infectious Disease Epidemiology & Prevention, Statens Serum Institut, Copenhagen, Denmark*
2. *Department of Infectious Disease Epidemiology, Norwegian Institute of Public Health, Oslo, Norway*
3. *Department of Health Security, National Institute for Health and Welfare, Helsinki, Finland*
4. *Department of Monitoring and Evaluation, Public Health Agency of Sweden, Solna, Sweden*
5. *Section for Geography, IGN, University of Copenhagen, Copenhagen, Denmark*
6. *Weather and Climate Change Impact Research, Finnish Meteorological Institute, Helsinki, Finland*
7. *Department of Hydrology, Norwegian Water Resources and Energy Directorate, Oslo, Norway*
8. *Global Health Section, Department of Public Health, University of Copenhagen, Copenhagen, Denmark*

* Corresponding author:

Katrin Gaardbo Kuhn

Infectious Disease Epidemiology & Prevention, Statens Serum Institut, Artillerivej 5, Copenhagen, Denmark

Mail: [kuh@ssi.dk](mailto:kuh@ssi.dk), Tel: +45 32688183

**Supplementary Methods**

To account for the large amount of zero counts in the data and identify which model best described the relationship between *Campylobacter* cases and climate, we explored using three modelling approaches based on Poisson regression: (1) standard Poisson regression, (2) zero-inflated Poisson regression and (3) standard Poisson regression omitting all observations with zero *Campylobacter* cases. The fit of each modelling approach was tested on a random sample of 10% (n=16,400) of the data records which had been omitted from the initial data analysis. The model which best predicted the omitted data records (number of cases per week in a given municipality) was the standard Poisson regression.

The Poisson regression is a generalized linear model type of regression analysis for modelling counts or rates which takes the form:

$$\log\left( \lambda i \right)=\beta0+\beta1xi1+\beta xi2+\beta3xi3\ldots$$

where λi is the number of *Campylobacter* cases per week in municipality *i,* and indeces 1,2,3… are climatic variables (i.e. temperature and precipitation) the preceding week.

Two models were developed: one for the ‘winter’ months (October – March) and one for the ‘summer’ months (April – September), and the respective models were used to predict *Campylobacter* cases per week per municipality in each season. To account for the fact that the number of *Campylobacter* cases varies in time and space, we offset by year, week and municipality. Offset variables are used in Poisson regressions where count outcomes (number of cases) reflect variation at different exposure levels. We aimed to reduce collinearity between climate variables by following a stepwise regression approach. The panel structure of the data (i.e. number of *Campylobacter* cases measured over multiple time periods and in multiple locations) was accounted for by using the random effects for counts syntax in the STATA statistical software.
